# Personalized Motor-Cognitive Exergame Training in Chronic Stroke Patients—A Feasibility Study

**DOI:** 10.3389/fnagi.2021.730801

**Published:** 2021-10-20

**Authors:** Simone K. Huber, Jeremia P. O. Held, Eling D. de Bruin, Ruud H. Knols

**Affiliations:** ^1^Physiotherapy and Occupational Therapy Research Center, Directorate of Research and Education, University Hospital Zurich, Zurich, Switzerland; ^2^Department of Health Science and Technology, Institute of Human Movement Sciences and Sport, Swiss Federal Institute of Technology, ETH Zurich, Zurich, Switzerland; ^3^Vascular Neurology and Neurorehabilitation, Department of Neurology, University Hospital Zurich, University Zurich, Zurich, Switzerland; ^4^Division of Physiotherapy, Department of Neurobiology, Care Sciences and Society, Karolinska Institute, Stockholm, Sweden; ^5^Department of Health, OST-Eastern Swiss University of Applied Sciences, St. Gallen, Switzerland

**Keywords:** stroke, mobility, training, cognition, gaming, virtual reality, rehabilitation, feasibility

## Abstract

**Purpose:** Exergame training may be beneficial for improving long-term outcome in stroke patients. Personalized training prescription applying progression rules, is missing. We adapted a theory-based taxonomy for a rehabilitation approach using user-centered exergames. The aims were primarily to investigate the feasibility of this rehabilitation approach, and secondarily to evaluate its performance of personalizing training progression, as well as explore the effects on secondary outcomes.

**Methods:** Chronic stroke patients (≥ 18 years) were included, who were able to walk 10 meters and stand for 3 min. The rehabilitation approach was administered twice per week for 8 weeks. As primary outcome, feasibility was evaluated by comparing achieved rates of inclusion, adherence, compliance, attrition, motivation, and satisfaction to pre-defined thresholds for acceptance. Secondary outcomes were (1) perceived motor and cognitive task difficulty throughout the intervention; (2) measures collected during baseline and post-measurements—a gait analysis, the Timed-up-and-go test (TUG), several cognitive tests assessing attentional, executive, and visuospatial functions.

**Results:** Thirteen patients [median: 68.0 (IQR: 49.5–73.5) years, median: 34.5 (IQR: 12.25–90.75) months post-stroke] were included, of whom ten completed the study. Rates for inclusion (57%), adherence (95%), compliance (99%), motivation (77%), and satisfaction (74%) were acceptable, however, the attrition rate was high (23%). The perceived motor and cognitive task difficulty predominantly moved below the targeted range. We found a significant change in the TUG (*p* = 0.05, *r* = 0.46) and medium-to-large effect sizes (*p* > 0.05) for swing time of the affected leg, the asymmetry index, time needed for the Trail-making test (TMT) A and accuracy for the TMT B and the Mental Rotation Test (MRT; 0.26 ≤ r ≤ 0.46).

**Discussion:** The intervention was feasible with minor modifications necessary, which warrants a larger trial investigating the effects of the rehabilitation approach following the adapted taxonomy on mobility, gait and cognitive functions. Two main limitations of the rehabilitation approach were; (1) the taxonomy decoupled motor and cognitive progression, which may be improper as motor and cognitive learning is coupled; (2) separate subjective ratings were used to guide the progression. Future studies should develop an instrument to objectively assess motor-cognitive task difficulty for monitoring the progression of an exergame-based training.

## Introduction

Stroke is a dominant global health burden and a major cause of long-term disability in adults (Feigin et al., 2016; Katan and Luft, 2018; Benjamin et al., 2019; Johnson et al., 2019). It can cause motor and cognitive impairments, depending on the involved brain region (Sun et al., 2014; Ursin et al., 2019). Despite carefully considered rehabilitation programs in the months following a stroke, full recovery is achieved only in a small proportion of stroke survivors (Gadidi et al., 2011). The majority of them, however, seem to reach a recovery plateau 6 months after stroke, which is typically at a lower functional level than before the stroke (Gadidi et al., 2011; McKevitt et al., 2011; Bernhardt et al., 2017). Intensive rehabilitation programs are then often terminated, leaving patients with residual impairments (Page et al., 2004; McKevitt et al., 2011). This can manifest by reduced mobility, gait functioning and cognitive ability, which limits stroke survivors in their independence and reduces quality of life (Mayo et al., 2002, 2009; De Wit et al., 2017). A further problem in chronic stroke survivors is physical deconditioning (Smith et al., 2012; Lennon et al., 2021), which increases the risk for secondary cerebrovascular diseases (Boulanger et al., 2018; Johnson et al., 2019; Lennon et al., 2021).

With intensive and continued rehabilitation, it is possible for chronic stroke patients to further regain and manage their impaired mobility, gait and cognitive functions (Ferrarello et al., 2011; Teasell et al., 2012; Lund et al., 2018; Cicerone et al., 2019). For continued rehabilitation interventions, physical exercise and cognitive training are recommended (Ferrarello et al., 2011; Gallanagh et al., 2011; Cumming et al., 2013). Different physical exercise regimes such as cardiorespiratory training, strength training, and neuromuscular training have improved mobility and gait in chronic stroke patients (Barak et al., 2016; Saunders et al., 2020). Moreover, physical activity has been found to improve cognitive functioning in chronic stroke (Oberlin et al., 2017). For cognitive rehabilitation, paper-and-pencil tasks and computerized cognitive training have been shown to beneficially affect specific cognitive abilities (e.g., working memory, attention and processing speed) (Yoo et al., 2015; Wentink et al., 2016; Cicerone et al., 2019). However, these programs often fail to show meaningful transfer effects to other cognitive domains or to daily-life tasks (Tiozzo et al., 2015; van de Ven et al., 2017). Recently, evidence has been emerging that approaches which combine motor and cognitive training, beneficially affect mobility, gait and cognitive functioning in older adults and neurological populations (Fritz et al., 2015; Levin et al., 2017; Stanmore et al., 2017; Mura et al., 2018). Such training approaches may, therefore, add to the possibilities to improve mobility, gait and cognitive outcome in chronic stroke (An et al., 2014; Tiozzo et al., 2015; Lee et al., 2017; Bo et al., 2019).

So-called Exergames are promising for simultaneous training of motor and cognitive functions (Herold et al., 2018). Exergames are active video games designed for a purpose beyond play (Michael and Chen, 2005; Rego et al., 2010), which require the trainee to perform whole-body movements (in contrast to finger movements in console gaming) to play the game and simultaneously challenge cognitive abilities (Herold et al., 2018). Their game-based character and the use of virtual reality (VR) can increase patients' motivation, therapy engagement and training intensity (Burdea, 2002; Shin et al., 2015; Swanson and Whittinghill, 2015; Johnson et al., 2016; Matallaoui et al., 2017; Zeng et al., 2017; Felipe et al., 2019; Lee et al., 2019). Exergames and VR methods in general were found to be feasible, safe and enjoyable in stroke patients, leading to higher adherence rates in rehabilitation interventions and may, thereby, promote improvements in functional outcome in the chronic stage after stroke (Iruthayarajah et al., 2017; Faria et al., 2018; Mura et al., 2018; Lee et al., 2019).

Personalization is important for rehabilitation programs, as it optimizes results and improves long-term adherence to the programs (Billinger et al., 2014). Exergames may be a powerful tool to personalize rehabilitation interventions, as they allow personalized therapy prescription according to the “FITT-VP” principles, including Frequency, Intensity, Time, Type, total Volume and Progression (Ruud et al., 2016; Medicine ACo et al., 2017). Nevertheless, a gap in current knowledge are schedules that enable such personalized therapy prescription for exergames, especially regarding personalized progression (Borghese et al., 2013). First steps to address this gap have been done using ‘Gentile’s taxonomy of motor learning' as a template for creating a personalized rehabilitation program using exergames (Borghese et al., 2013; Wüest et al., 2014) (more detailed information on this can be found in section “Adaption of Gentile's Taxonomy”). “Gentile's taxonomy of motor learning” is a systematic classification categorizing motor skills and movement according to two general dimensions of actions (Gentile, 1987; Laguna, 2008). So far, however, applications of this taxonomy were restricted to tailored progression and variability of motor tasks. As exergames are integrated motor-cognitive trainings and also cognitive training should be carefully and personally prescribed (Herold et al., 2018; Cicerone et al., 2019), it can be hypothesized that additionally considering cognitive tasks for the personalized progression may add further benefits in the long-term rehabilitation of chronic stroke patients. We, therefore, adapted “Gentile's taxonomy of motor learning” by adding a third cognitive dimension using customized and purpose-centered exergames.

The primary aim of this study was to examine the feasibility of this adapted taxonomy for use in the rehabilitation of mobility, gait, and cognitive functions in chronic stroke patients. We assumed that application of the adapted taxonomy would result in acceptable inclusion, adherence, compliance and attrition rates and that motivation and satisfaction with the training would be and stay high throughout the intervention period. As secondary aims, we (1) assessed whether applying the adapted taxonomy would ensure personalized perceived task difficulty throughout the intervention; and (2) wanted to gain first insight into possible effects of the rehabilitation approach on mobility, gait and cognitive functions in chronic stroke patients.

## Materials and Methods

### Study Design and Procedures

**Figure 1:**
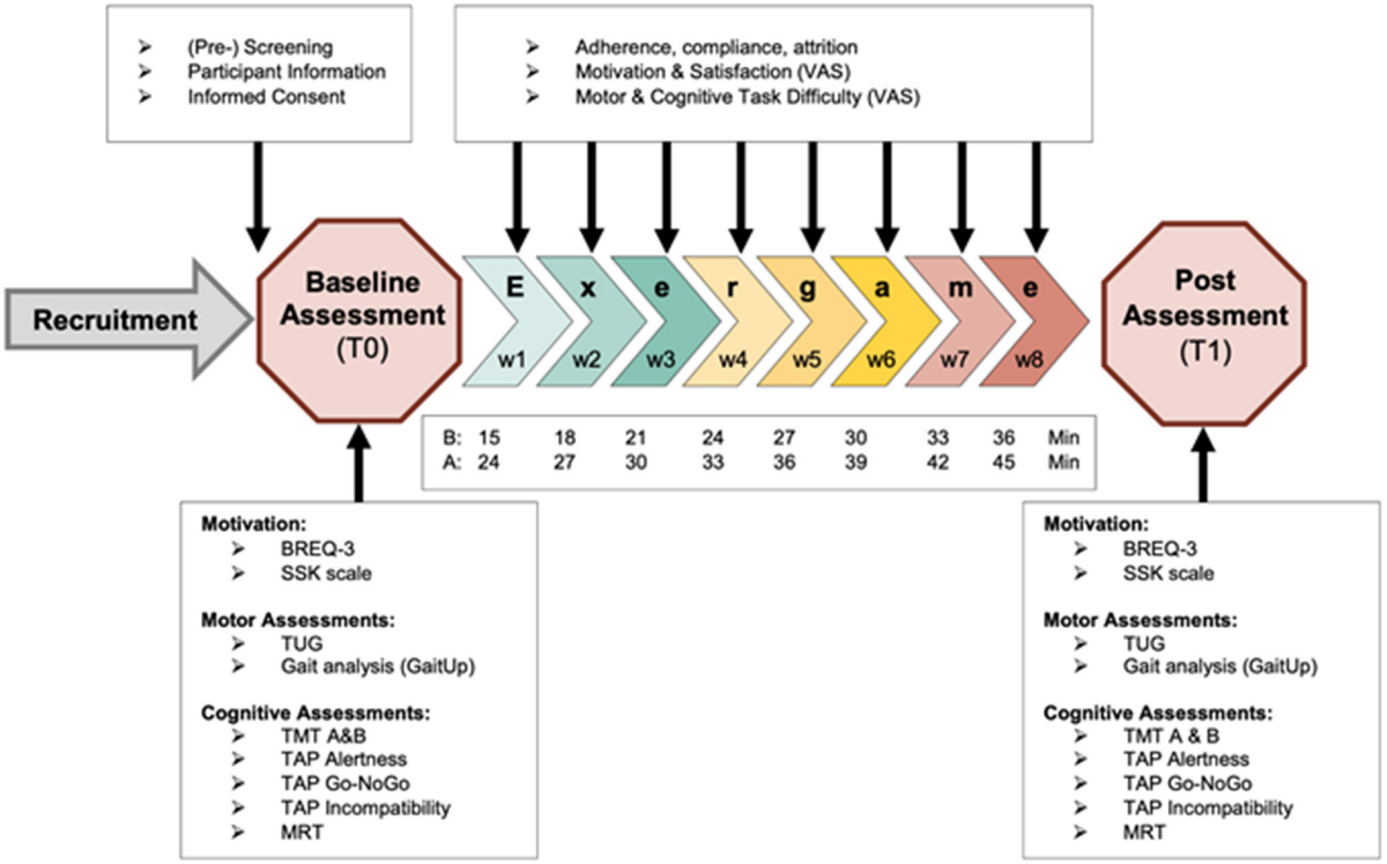
Study flowchart, shows all study procedures and intervention details. B, basic training group; A, Advanced training group; Min, minutes of training in the respective week; VAS, visual analog scale; BREQ-3, Behavioral Regulation in Exercise Questionnaire; SSK, Sport- and Movement-specific Self-Concordance scale; TUG, Timed-up-and-go test; TMT, Trail making test; TAP, Test of attentional performance; MRT, mental rotation test.

This study was a feasibility trial. The recruitment was facilitated by a physiotherapy center and a senior home in the Canton Zürich (Switzerland). Instructed physiotherapists and care staff pre-screened the patients and older adults at the study sites on having suffered a stroke and interest in a study participation and if so, made contact to the researchers. Participants were then screened on all eligibility criteria face-to-face or by phone call (see section “Participants and Sample Size Considerations” for a more detailed description) by trained movement scientists of the study team. If meeting all criteria, participants were informed about the study procedures, benefits, and risks and subsequently, if willing to participate, signed informed consent and underwent baseline measurements. Depending on their baseline time for the timed-up-and-go test, participants were assigned either to a basic or to an advanced training group. Participants who completed the test in more than 10 s were assigned to the basic group, while participants who needed 10 s or less were assigned to the advanced group (Bohannon, 2006). The intervention lasted 8 weeks and sessions were performed twice per week for 15–45 min. All sessions were performed on separate days of the week and in case possible, with at least one resting day between sessions (e.g., always on Monday and Wednesday). The session duration was progressed over time, adding 3 min of intervention after every week. The basic group started with 15 min of training in the first week and ended with 36 min in the last week, while the advanced group went from 24 to 45 min. This meets the lower border of recommendations for exergame and VR interventions in stroke patients and healthy older adults (Lee et al., 2019; Stojan and Voelcker-Rehage, 2019). As motor learning and cognitive functions were the target of the intervention, the intensity of the trainings was monitored by perceived task difficulty and guided by the adapted taxonomy (see section Adaptation of Gentile's Taxonomy). The study procedures are summarized in the Study Flowchart (Figure 1). This study was approved by the Ethical Committee of the ETH Zürich (approval nr. 2019-N-180).

### Participants and Sample Size Considerations

Participants had suffered a stroke at least 6 months ago, and were adult (≥ 18 years), able to walk ten meters as well as stand for 3 min without assistance and German speaking. Participants were excluded if they suffered from a neglect, a hemi-anopsia, other neurological diseases or other progressive and uncontrolled diseases. Additionally, as this study was conducted during the COVID-19 pandemic, further eligibility criteria regarding high risk for a serious course of disease had to be complied with (acute, rapidly progressing or terminal illnesses, chronic respiratory disease, therapy or status weakening the immune system, cancer, adipositas, BMI ≥ 40). A sample size of 12 or more participants was targeted as for pilot and feasibility trials, at least 12 participants are recommended (Julious, 2005; Moore et al., 2011).

### Exergame Device

**Figure 2:**
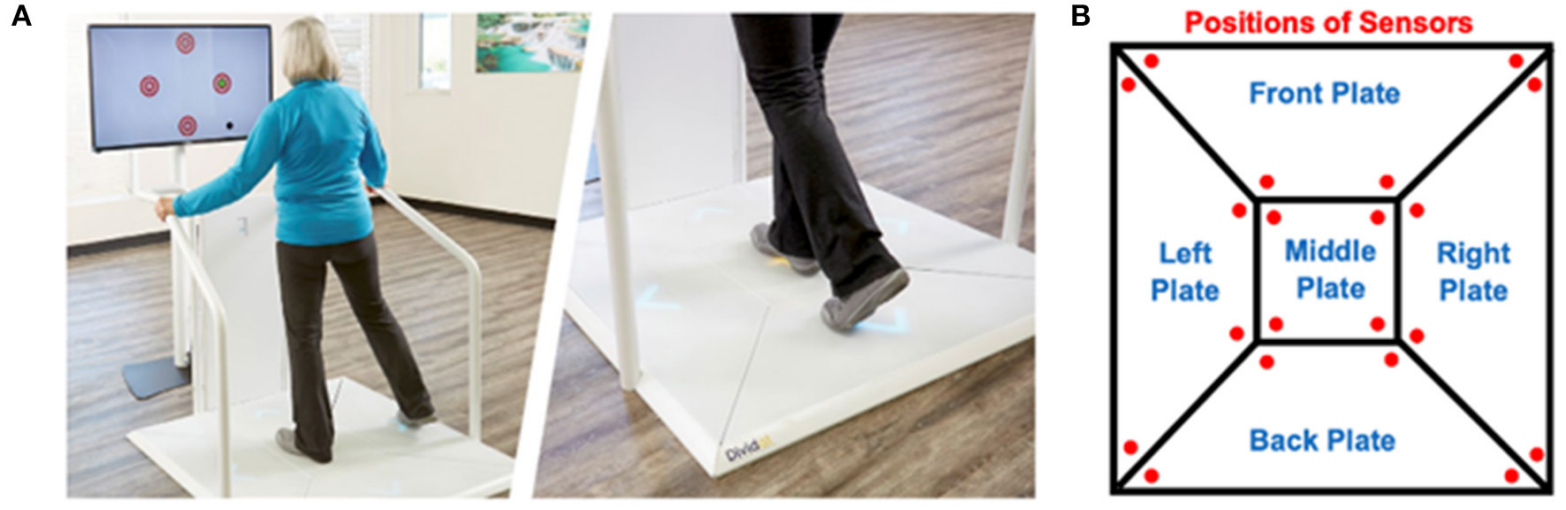
**(A)** A participant training on the Dividat SENSO (Dividat AG, Schindellegi, Switzerland). **(B)** Top view of the pressure-sensitive plate of the Dividat SENSO, showing the position of the sensors.

The Dividat SENSO (Dividat AG, Schindellegi, Switzerland) exergame device, which consists of a pressure-sensitive plate and screen, was used. Trainees perform body weight shifts and step-based movements on the plate, which are used to control the cognitively demanding video games presented on the screen in front of them (Figure 2A). The plate is divided into five sub-plates, under which twenty sensors are placed to detect information on center of pressure position and movement timing (Figure 2B). This information is used by the system to provide real-time feedback to the gamer's performance. Visual and auditory feedback is given by the games on the screen, while the sub-plates vibrate to additionally give tactile feedback and pronounce the visual and auditory feedbacks. This enables the participant to interact with the video games. A selection of games is available, each of which is specified and graded for different motor and cognitive functions. The plate is equipped with a handrail on three sides to ensure safety of the participant and prevent falls (Figure 2A).

### Adaptation of Gentile's Taxonomy

The rehabilitation approach was based on ‘Gentile’s taxonomy of motor learning' (Gentile, 1987; Laguna, 2008), which is a skill progression platform structured along two dimensions with four sub-dimensions each: environmental context (stationary vs. in-motion scheme and inter-trial variability vs. no inter-trial variability) and action function (body stability vs. body transport each with and without object manipulation), resulting in 16 motor skill categories (Laguna, 2008). This two-dimensional structure can easily be adapted to training using virtual environments. For patients with low skill levels an environment can be designed with low action task constraints and no distracting features in the virtual environment. When a game under these initial conditions is performed well, the environment, the action function, or both can be manipulated and made more complex. This enables stepwise progression through the framework. Used this way Gentile's taxonomy provides an “ecologically valid” practical and daily-life relevant way of motor learning. Due to the properties of the exergame device, the sub-dimensions “stationary vs. in-motion” as well as “without object manipulation vs. with object manipulation” were not distinguished within the adapted taxonomy for this study.

**Figure 3:**
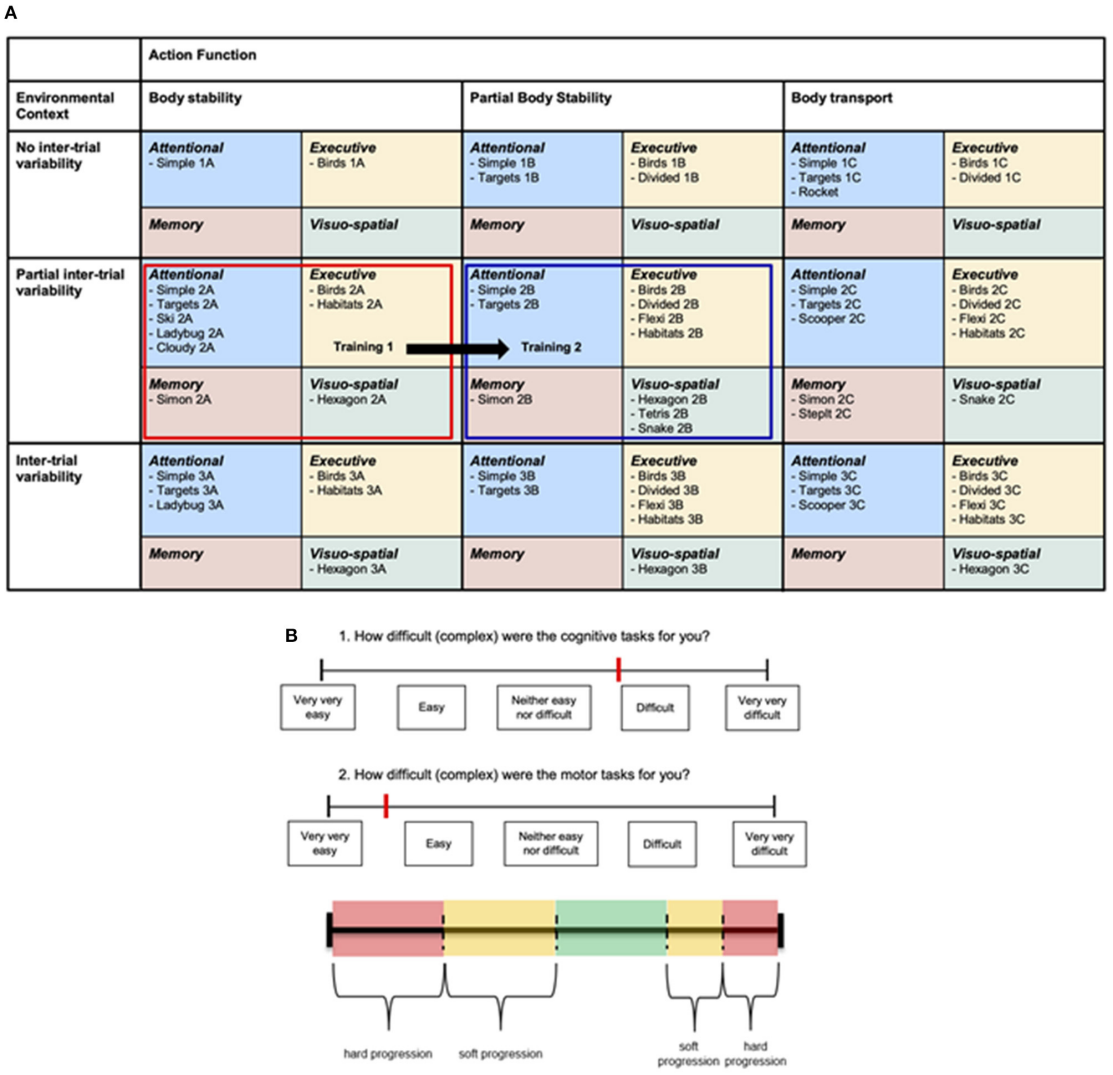
**(A)** The final adapted taxonomy and two “position squares” of a first and second training. **(B)** Visual analog scale, which were used to rate perceived motor and cognitive task difficulty after each training session, and the scale indicating the ranges for no (green), soft (yellow), and hard (red) progression. Two possible rating locations are indicated (red lines), which would lead to the shift of the red “position square” of the first training to the green “position square” for the second training in **(A)**.

The adaption was performed in two steps. (1) We added one sub-dimension to both axes (environment, action) to provide variability in levels and to fully exploit the variety in possible motor and cognitive tasks of the Dividat SENSO. This defined three sub-dimensions for the environmental context (no inter-trial variability, partial inter-trial variability, inter-trial variability) and three sub-dimensions for the action function context (body stability, partial body transport, body transport). For the latter, we defined the three sub-dimensions as follows (see also Table 1); (1) body stability contained tasks during which the center of pressure (COP) moved within stable limits of stability; (2) in partial body transport, the COP moved within moderately changing limits of stability; and (3) in body transport, the COP moved within constantly and rapidly changing limits of stability. This resulted in nine motor skill categories with different levels regarding “body stability vs. body transport” and “no inter-trial variability vs. inter-trial variability” (Table 1). (2) For adding a cognitive dimension, we further sub-divided each of these motor skill categories into four sub-categories; one for each of four different cognitive domains, which are targeted with the available games (attention, executive, memory, and visuo-spatial functions). Finally, the adapted taxonomy consisted of 36 skill sub-categories, which offered different motor skill levels in combination with different cognitive challenges (Figure 3A). Due to limited possibilities to adapt all games to all skill categories and a lack of games targeting the domain memory, some of these 36 skill sub-categories remained empty for this study (Figure 3A).

**Table 1:** Adapted taxonomy with 9 motor skill categories (after step 1 of the adaptation).

	**Action function**		
**Environmental context**	**Body stability**	**Partial body transport**	**Body transport**
No inter-trial variability	1A	1B	1C
	Body-weight shifting, small steps with handrail Fixed stimuli sequence, fixed interval between stimuli	Small and wide steps without handrail, Squats, Lunges Fixed stimuli sequence, fixed interval between stimuli	Step Touches, Walking, Dribbling, Jumps, Airex Fixed stimuli sequence, fixed interval between stimuli
Partial inter-trial variability	2A	2B	2C
	Body-weight shifting, small steps with handrail Random stimuli sequence, fixed interval between stimuli	Small & wide steps without handrail, Squats, Lunges Random stimuli sequence, fixed interval between stimuli	Step Touches, Walking, Dribbling, Jumps, Airex Random stimuli sequence, fixed interval between stimuli
Inter-trial variability	3A	3B	3C
	Body-weight shifting, small steps with handrail Random stimuli sequence, adaptive interval between stimuli	Small & wide steps without handrail, Squats, Lunges Random stimuli sequence, adaptive interval between stimuli	Step Touches, Walking, Dribbling, Jumps, Airex Random stimuli sequence, fixed interval between stimuli

To comply with the training principles of personalized intensity and progression [FITT-VP principles for training prescription (Ruud et al., 2016; American College of Sports et al., 2018)], we developed progression rules guided by subjective ratings of the “perceived task difficulty” (Bratfisch, 1972; Paas et al., 2003; Sweller et al., 2011), rated on a visual analog scale (VAS) from 0 (“very very easy”) to 9 (“very very difficult”) after each training session (Figure 3B) by the participants. These ratings were used to propose the next training session. Based on the “Cognitive Load Theory,” we targeted a task difficulty ranging from “neither easy nor difficult” to “difficult” (Sweller, 2011). Therefore, a rating within this range resulted in no progression, meaning no shift of the “position square” (compare section Intervention/Personalized Exergame Training) before the next training. Was the rating lower than the target range, either a soft progression (Figure 3B, yellow range) or a hard progression (Figure 3B, red range) was administered, increasing the level by moving to the right and to the bottom of the table, respectively. A soft progression moved the “position square” one sub-category and a hard progression moved it two sub-categories. In case of a rating higher than the target range, the soft or hard progression was administered in opposite direction, decreasing the level of difficulty. The location of the cognitive rating guided the progression on the y-axis of the taxonomy, while the motor rating was used to guide the progression on the x-axis (see example Figure 3B).

### Intervention/Personalized Exergame Training

A “position square” defined both motor skill difficulty levels and cognitive content of each training session (see Figure 3A, e.g., red square). Each training session consisted of a 3-min warm-up, a main part (9–39 min depending on group assignment and time point in the intervention) and a 3-min cool-down. Both, the warm-up and cool-down games were chosen from the top-left sub-category within the “position square,” as this contained the games with the lowest motor and cognitive levels according to the taxonomy. The warm-up and cool-down games should provide a motivating start and a feeling of success at the end of the session, respectively. The main part of the training was composed of games from the remaining three sub-categories, which lasted between 60 and 180 s each. The games were arranged so that sufficient variability was provided and that the perceived task difficulty increased after the warm-up, reached a peak in the last third of the main part and then decreased again toward the cool-down. Participants could take breaks or end the training session at all times, if the sessions were too long, if they were overcharged with the tasks or if they felt they did not intend to continue. All training sessions were supervised one-to-one by trained movement scientists of the study team and took place face-to-face in the physiotherapy center and senior home.

### Measures

All data collection including primary and secondary outcomes was performed by trained movement scientists of the study team and took place face-to-face in the physiotherapy center and senior home. As primary outcome, the feasibility data were collected during the screening process and the training sessions (see **Table 3**). Regarding adherence, sixteen training sessions were planned for all participants. In case a session had to be canceled, it was replaced if possible, which could lead to a moderate extension of the 8 weeks intervention period. To determine compliance, completed training time was divided by the total planned training time, which was defined by the group assignment (basic or advanced) and the time point in the intervention (see section Study Design and Procedures). As participants could end training sessions earlier, completed training time could be lower than planned training time. To assess motivation and satisfaction with the trainings, participants rated their motivation and satisfaction on visual analog scales ranging from 0, “Not all motivated/satisfied” to 10, “Totally motivated/satisfied” collected after each training session (Supplementary Material 1).

To further explore the performance of the taxonomy and progression rules, the secondary outcomes included the VAS ratings of the perceived motor and cognitive task difficulty and the individual trajectories through the taxonomy of the participants. Further secondary outcomes were motor and cognitive assessments conducted during the baseline and post-measurements to gain a first insight into possible training effects. Two movement scientists were present during each measurement session, one of them performing the assessments and the other one securing the participant during upright activities to prevent falls. A gait analysis was conducted using Physilog® sensors (Gait up Sàrl, Lausanne, Switzerland), which have been shown to deliver valid and reproducible results for spatiotemporal gait analysis in stroke patients (Lefeber et al., 2019). The assessment followed the protocol for the “*Figure-of-8 Walk Test”* (F8W*)* by Hess et al. (2010), which is feasible and meaningful in stroke patients (Wong et al., 2013). Participants completed one F8W according to the protocol, while time was measured with a stopwatch. Then, the Physilog® sensors were switched on and the participants walked five F8Ws in a row to measure spatiotemporal gait parameters during straight and curved steady state walking. This procedure (1x F8W; 5x F8W) was repeated three times to capture at least fifty gait cycles, which is recommended for a reliable assessment of spatio-temporal gait parameters (Konig et al., 2014). From the output of the Physilog® gait analysis, values on “time needed for one F8W,” gait speed, asymmetry index (based on stride time), stride length variability, stride time variability, double support time, swing time, and swing width were collected. A Timed-up-and-go test (TUG) was conducted to measure functional mobility, following methods described previously (Ng and Hui-Chan, 2005)^1^. The participants performed the test three times, and the scores were then averaged. The TUG has been shown to be feasible, reliable and valid in stroke patients (Ng and Hui-Chan, 2005).

Cognitive outcomes included the Trail-making-test A and B [TMT A & B (Reitan and Skills, 1958; Bowie and Harvey, 2006)], the Mental-rotation-test [MRT, “Shepard and Metzler paradigm” (Shepard and Metzler, 1971)] and three sub-tests of the Test of Attentional Performance [TAP (Zimmermann and Fimm, 1992)], namely (i) Alertness test, (ii) Go-NoGo test and (iii) Incompatibility test. The TMT A and B are sub-tests to assess the general information-processing speed and executive functions, particularly mental flexibility, respectively. The TMT was found to be a valid and reliable test of these functions (Reitan and Skills, 1958; Wagner et al., 2011). The MRT is a measure of visuo-spatial skills, particularly the ability for mental rotation (Shepard and Metzler, 1971). The TMT and the MRT were conducted using a computer with PEBL software (Mueller and Piper, 2014). The TAP allows an appropriate and differentiated diagnosis of attentional deficits and the used sub-tests provide data on the simple reaction time, selective attention and inhibition as well as the reaction to an incompatible stimulus (Zimmermann and Fimm, 1992). The subtests of the TAP have been widely used in various populations including stroke survivors (Starovasnik Zagavec et al., 2015; Spaccavento et al., 2019).

Motivation for physical activity was assessed at baseline and post measurements to observe the influence of the exergame training on general motivation for being active. Participants answered the Behavioral Regulation in Exercise Questionnaire [BREQ-3, German version of the BREQ-2 (Mullan et al., 1997; Markland and Tobin, 2004)] and the Sport- and Movement-specific Self-concordance scale [SSK (Fuchs et al., 2005)]. Both assess the degree of self-determination in terms of exercise behavior. However, while the BREQ-3 covers motivational aspects for physical activity in general, the SSK is directed into the future and, therefore, provides information about the future intention toward physical activity. The analysis of the BREQ-3 and the SSK scale leads to scores for different sub-types of motivation, which can be used to evaluate the degree of self-determination participants have toward physical activity. Intrinsic, integrated and identified motivation represent different forms of self-determined motives for physical activity, while introjected and external motivation are linked to less self-determined motives (Markland and Tobin, 2004; Fuchs et al., 2005; Chemolli and Gagne, 2014). The BREQ-3 additionally investigates amotivation, which can be used to explain low ratings in other motivational domains (Markland and Tobin, 2004). For the SSK scale, the SSK index was additionally calculated, which is a summary score integrating all motivation subtypes. A higher SSK index stands for higher self-determination in physical activity and vice versa (Fuchs et al., 2005).

### Feasibility Analysis

The feasibility of the rehabilitation approach was determined using a pre-defined feasibility protocol including six parameters with thresholds (see Supplementary Material 2). The feasibility criteria were defined to detect problems with the recruitment process (inclusion rate), the intervention itself (attrition and satisfaction rates), the frequency and time applied (adherence, compliance and attrition rates), as well as the personalized progression (adherence, compliance, motivation and satisfaction rates). Thresholds for the feasibility criteria were based on guidelines for inclusion (≥50%), adherence (≥80%), compliance (≥80%), and attrition (≤15%) (Nyman and Victor, 2011) and established from results from comparable studies for motivation (≥60%) and satisfaction (≥60%) (Bernardoni et al., 2019; Spildooren et al., 2019; van Beek et al., 2019). The feasibility protocol contained instructions on how to interpret the results in all possible scenarios according to the different feasibility outcomes as well as on how to continue with the rehabilitation approach in a subsequent study (Thabane et al., 2010).

### Analysis of Secondary Outcomes

The evaluation of the perceived motor and cognitive task difficulty and the individual trajectories was performed to discover reasons why the rehabilitation approach did or did not achieve personalized progression of task difficulty. Therefore, the VAS ratings of perceived motor and cognitive task difficulty were averaged for each session and compared to a pre-defined target range of perceived task difficulty (Sweller, 2011). This and the exploration of the individual trajectories of each participant should give in-depth insight in the development of the perceived task difficulty and reveal over- and undercharging of the participants. The data of the remaining secondary outcomes were reported and analyzed non-parametrically (Field, 2013), therefore descriptive data were reported and represented as median and interquartile range. Within-subject changes from baseline to post measurements were analyzed with the Wilcoxon signed-rank test. Median differences with interquartile range, the test statistics T, p-values and effect sizes were provided for each outcome. As recommended for pilot feasibility trials the main emphasis of our trial was placed on feasibility and not on statistical significance (Thabane et al., 2010). Thus, due to the exploratory nature of the analysis for the secondary outcomes in this study, no correction for multiple comparisons was performed (Rothman, 1990; Bender and Lange, 2001; Althouse, 2016). Consequently, *p*-values must be interpreted with caution. The level of statistical significance was set to *p* < 0.05. Effect sizes were interpreted to be small (*r* < 0.30), medium (0.30 ≤ *r* < 0.5) and large (*r* ≥ 0.50) (Fritz et al., 2012). All statistical analyzes were performed using SPSS Statistics (version 26 for windows; IBM, Chicago, IL, USA).

## Results

### Participants Overview

**Figure 4:**
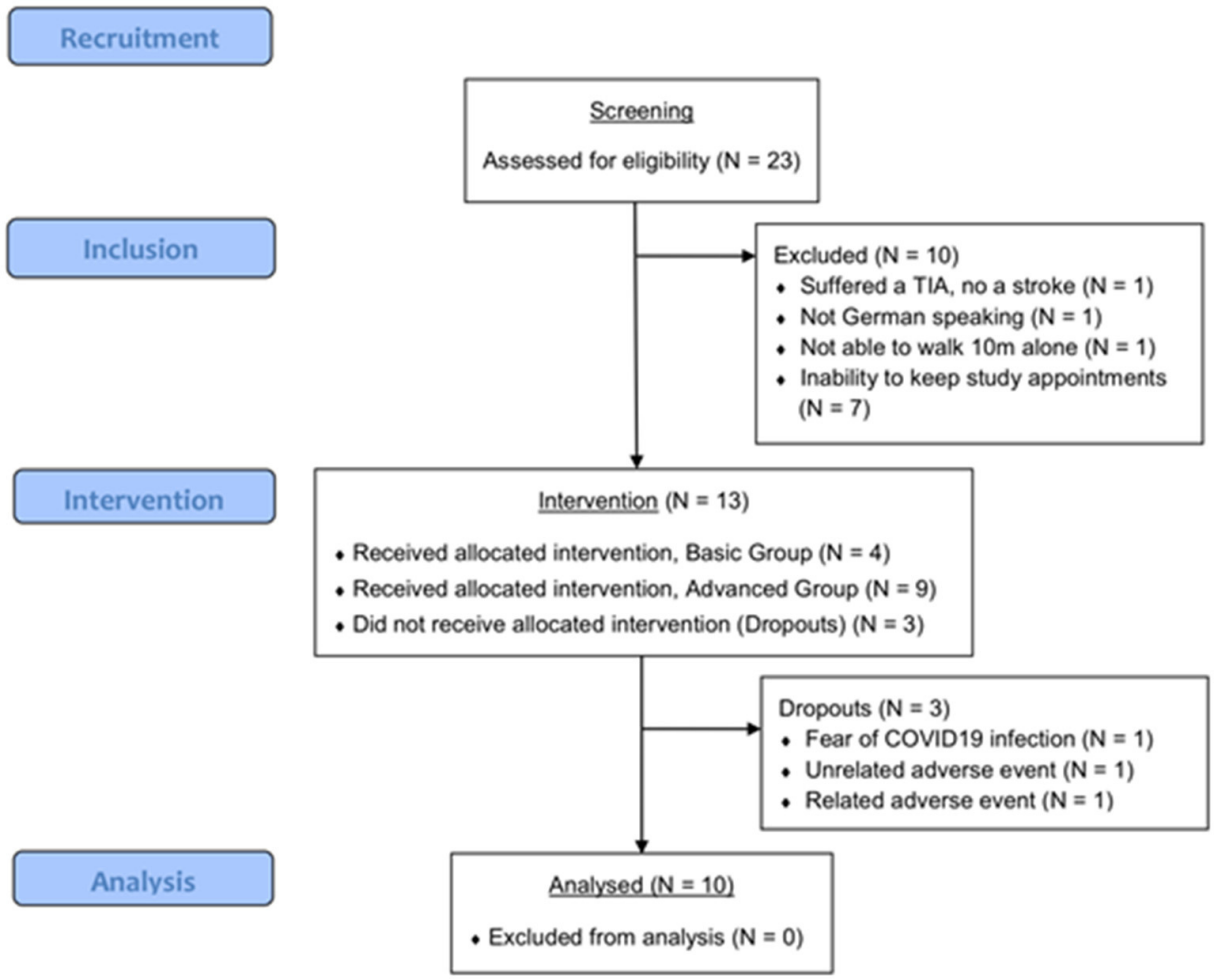
CONSORT diagram of the participant flow.

Twenty-three possible participants were screened in March 2020 and from September to October 2020 of whom 13 were included (Figure 4). After having started recruiting, the study had to be postponed between April and August 2020 because of a national shutdown due to the COVID19 pandemic. The most common reason for non-inclusion was the inability of the participants to come to the study centers for the training sessions (Figure 4). Additionally, one screened patient had suffered a transient ischemic attack and was therefore excluded. Twelve participants of the included participants were recruited from the physiotherapy center and one participant lived in the senior home. This entailed that the majority (12 out of 13) of the participants received regular physical therapy (1 or 2 sessions of 30–60 min) beyond the training sessions of this study. Of the 13 initially included participants, 10 completed post-measurements while three dropped out of the study earlier. The reasons for the dropouts were the following; one participant wanted to stop the study participation due to fear of COVID19 infection at the study center; one participant suffered a severe flu (adverse event unrelated to the intervention) and was absent for several weeks, which made a continuation of the training impossible; one participant experienced an uncomfortable worsening of their head tremor after the first two trainings (adverse event related to the intervention), which forced them to stop the intervention. The participant flow is shown in Figure 4 and baseline characteristics are presented in Table 2.

**Table 2:** Baseline characteristics.

	**Median**	**IQR**	**Range**	***N***	**%**
Age [years]	68.0	(49.5, 73.5)	34–79		
Gender [women/men]				5/8	38.5/61.5
Time post-stroke [months]	34.5	(12.25, 90.75)	6–179		
Impaired body side ^*^ [right/left]				4/9	30.8/69.2
FAC [ranging 0-5, 2/4/5]	5	(5, 5)	2–5	1/1/11	7.7/7.7/84.6
TUG [s, >10s/ ≤ 10s]	8.24	(6.78, 11.90)	7.50–29.03	4/9	30.8/69.2
Walking aids [none/one cane]				12/1	92.3/7.7
Education [years]	12.0	(12.0, 17.5)	9 - 20		
School level [Secondary, higher]				3/10	23.1/76.9
TMT A [s]	32.20	(28.26, 37.92)	23.54–85.26		
TMT B [s]	51.58	(38.90, 76.77)	24.08–289.90		

*IQR, Interquartile Range; N, number of participants; %, percentage of participants*.

* *For two participants the impaired body side was unclear; therefore the body side with greater stride length variability was assumed to be the impaired body side*.

### Feasibility

**Figure 5:**
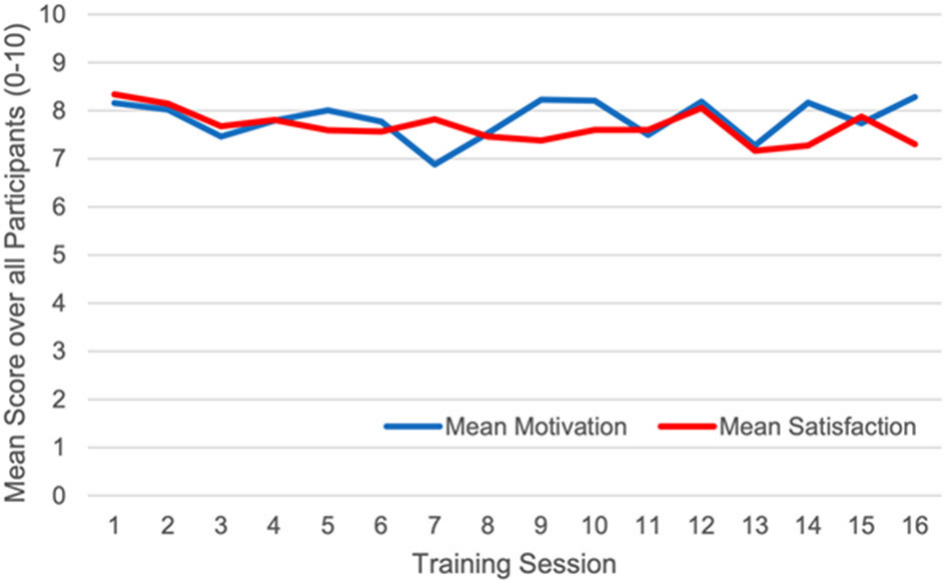
Mean motivation and satisfaction ratings over all participants.

We found acceptable rates of inclusion (57%), adherence (95%), compliance (99%), motivation (77%), and satisfaction (74%), of which the highest was the compliance rate with almost full completion of planned training time (Table 3). The attrition rate (23%), however, was higher than the pre-study defined threshold set for acceptance. Mean motivation and satisfaction were rated “rather high” to “high” over the whole course of the intervention (Figure 5).

**Table 3:** Feasibility results.

**Feasibility criteria**	**Threshold (%)**	**Data**	**Results**	**Outcome (%)**	**Criteria met**
Inclusion rate	≥ 50^a^	Screened participants [#] Included participants [#]	23 13	57	Yes
Adherence rate	≥ 80^a^	Total offered training sessions [#] Total attended training sessions [#]	189 165	95	Yes
Compliance rate	≥ 80^a^	Total offered training time [min] Total attended training time [min]	4,974 4,915	99	Yes
Attrition rate	≤ 15^a^	Included participants [#] Dropouts [#]	13 3	23	No
Motivation rate	≥ 60^b^^,^^c^^,^^d^	Mean VAS rating over all sessions Maximal VAS rating	7.67 10.0	77	Yes
Satisfaction rate	≥ 60 ^b^^,^^c^^,^^d^	Mean VAS rating over all sessions Maximal VAS rating	7.44 10.0	74	Yes

a *Nyman and Victor (2011)*.

b,c,d *Thresholds established based on results from comparable studies (Bernardoni et al., 2019; Spildooren et al., 2019; van Beek et al., 2019)*.

### Secondary Outcomes

**Figure 6:**
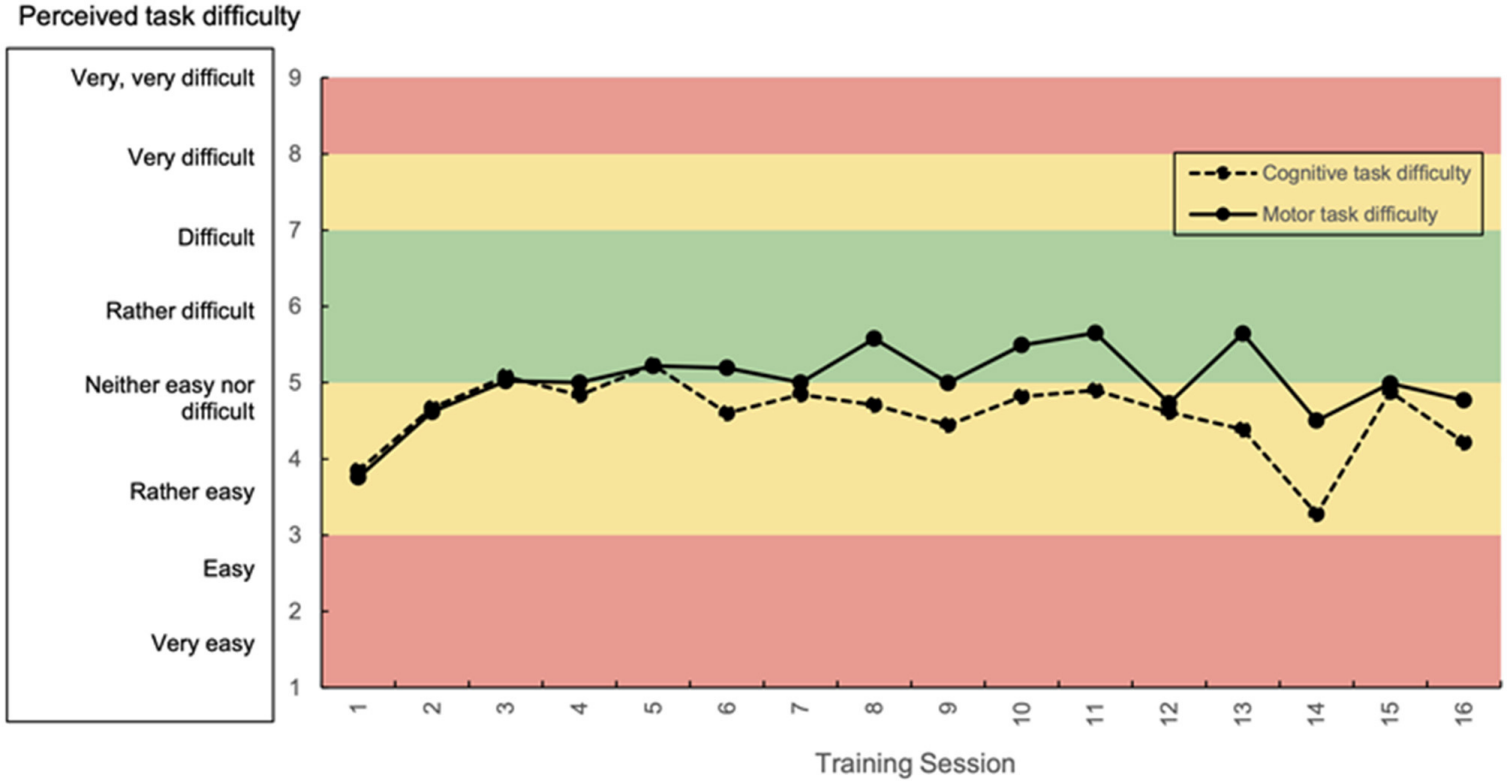
Mean ratings for perceived motor and cognitive task difficulty over all participants. Green area: targeted range of perceived task difficulty.

**Figure 7:**
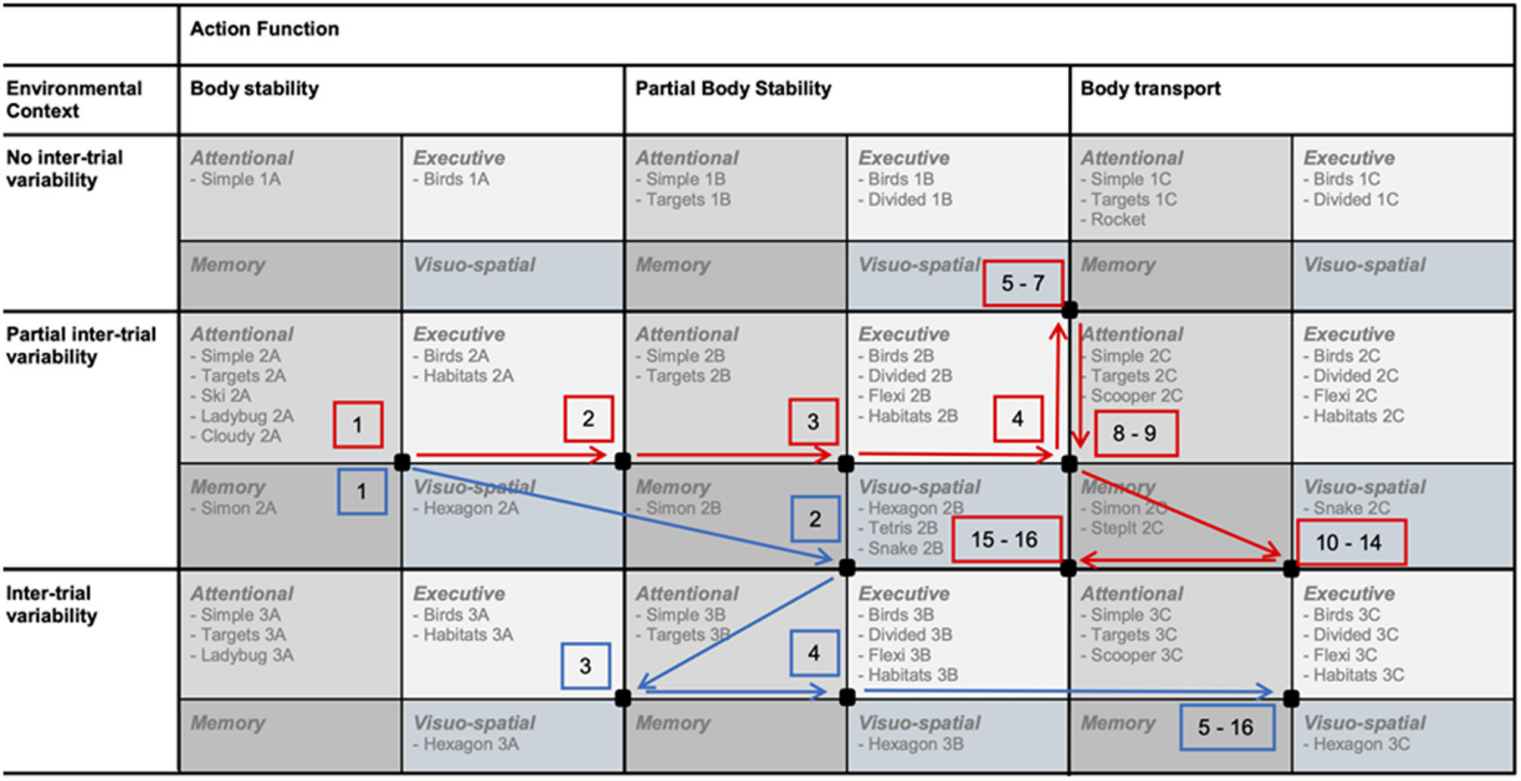
Trajectories of two participants (red, blue) showing their “path” through the taxonomy from the first to the sixteenth training session. Black dots represent the center of the respective “position squares”.

The mean perceived motor and cognitive task difficulty were rated from “rather easy” to “rather difficult” throughout the intervention period (Figure 6). The mean motor demand was generally rated higher than the mean cognitive demand. Participants moved in the taxonomy in individual ways (Figure 7 shows two example trajectories).

The parameters of the gait analysis remained similar from pre- to post-measurements and no meaningful changes were observed with predominantly small effect sizes (Table 4). The swing time of the affected leg showed a medium effect size (*p* = 0.21, *r* = 0.32) and the asymmetry index a small-to-medium effect size (*p* = 0.29, *r* = 0.26). The TUG improved significantly from pre- to post-measurements with a medium-to-large effect size (*p* = 0.05, *r* = 0.46, Table 4). None of the cognitive assessments showed changes from pre- to post-measurements, however, medium effect sizes were found for improvements in time needed for TMT A (*p* = 0.11, *r* = 0.35), in accuracy in the TMT B (*p* = 0.18, *r* = 0.30) and accuracy in the MRT (*p* = 0.15, *r* = 0.33, Table 5). Additionally, medium to large effect sizes were found for increased reaction times in the TAP incompatibility test for both, compatible (*p* = 0.12, *r* = 0.46) and incompatible (*p* = 0.25, *r* = 0.33) stimuli (Table 5). One participant was not able to perform the TMT with the computer mouse and therefore, the paper-and-pencil version was administered as an alternative. Differences in the number of participants who were included in the analyzes of the different assessments were caused by several reasons; (1) technical problems with the Physilog® sensors during one measurement (spatio-temporal gait analysis, Table 4); (2) inability of one participant to perform the motor assessments at the post measurements due to a temporary worsening of the physical condition (TUG and gait analysis, Table 4); and (3) inability of four participants to perform the cognitive tests with both hands (TAP Incompatibility, MRT, Table 5).

**Table 4:** Results secondary motor outcomes.

**Motor outcomes**	***N***	**T0**	**T1**	**Difference T1–T0**	**95% CI**	**T**	**Asymptotic significance**	**Effect size**
F8W mean [s]	9	9.79 (7.96, 12.81)	9.35 (8.16, 11.90)	−0.12 (−1.69, 1.32)	[−1.67, 1.27]	20.00	0.77	0.07
Gait speed [m/s]	8	0.80 (0.62, 1.13)	0.75 (0.52, 0.94)	0.01 (−0.17, 0.04)	[−0.31, 0.13]	17.00	0.89	0.04
Asymmetry index [%]	8	1.09 (1.02, 1.22)	1.11 (1.03, 1.24)	−0.01 (−0.04, 0.00)	[−0.11, 0.09]	10.50	0.29	0.26
Stride length var aff [%]	8	20.81 (15.86, 26.77)	19.23 (14.34, 24.11)	−1.89 (−5.66, 4.48)	[−7.90, 4.05]	14.00	0.58	0.14
Stride length var unaff [%]	8	21.16 (15.53, 25.04)	21.68 (16.70, 26.57)	1.95 (−5.09, 6.01)	[−4.89, 6.80]	20.00	0.78	0.07
Stride time var aff [%]	8	6.67 (5.02, 10.29)	4.46 (3.42, 9.85)	−0.88 (−5.27, 2.61)	[−4.52, 2.20]	14.00	0.58	0.14
Stride time var unaff [%]	8	6.84 (4.74, 9.21)	6.45 (3.78, 10.12)	0.44 (−1.83, 2.54)	[−2.26, 2.38]	21.00	0.67	0.11
Double support time [%]	8	27.45 (25.03, 32.81)	31.01 (26.06, 34.16)	0.09 (−1.10, 3.04)	[−2.18, 5.14]	22.00	0.58	0.14
Swing time aff [%]	8	37.20 (36.99, 40.04)	36.83 (34.81, 40.04)	−0.37 (−1.67, 0.06)	[−1.57, 0.16]	9.00	0.21	0.32
Swing time unaff [%]	8	34.42 (30.62, 37.83)	34.01 (29.15, 36.14)	0.16 (−1.56, 1.56)	[−3.89, 2.36]	19.00	0.89	0.04
Swing width aff [cm]	8	7.58 (4.05, 9.39)	5.82 (4.87, 9.21)	−0.17 (−2.27, 2.07)	[−1.91, 1.69]	16.00	0.78	0.07
Swing width unaff [cm]	8	3.73 (0.71, 7.53)	4.20 (1.64, 5.39)	0.35 (−1.89, 1.17)	[−2.15, 1.69]	16.00	0.78	0.07
TUG mean [s]	9	8.24 (6.78, 11.90)	7.23 (6.16, 9.27)	−1.01 (−1.67, 0.57)	[−2.17, −0.08]	6.00	**0.05**	0.46

*N, number of participants included in test; T0, Baseline, T1, Post-Measurements, CI, confidence interval; T, test statistic of Wilcoxon signed rank test; var, variability, aff, affected; unaff, unaffected*.

*Values displayed in median (interquartile range); [s] seconds, [m/s] meters per second, [m] meters; in bold, significant at p < 0.05*.

**Table 5:** Results secondary cognitive outcomes.

**Cognitive outcomes**	***N***	**T0**	**T1**	**DifferenceT1–T0**	**95% CI**	***T***	**Asymptotic significance**	**Effect size**
**TMT**								
TMT A time [s]	10	31.74 (27.43, 37.81)	30.16 (25.45, 31.66)	−3.67 (−5.44, 1.26)	[−5.07, 0.72]	12.00	0.11	0.35
TMT A accuracy	10	1.00 (0.99, 1.00)	0.96 (0.96, 1.00)	0.0 (0.96, 1.00)	[−0.65, 0.05]	5.00	0.50	0.15
TMT B time [s]	10	56.29 (40.81, 85.81)	59.61 (34.00, 69.28)	−7.44 (−29.08, 11.27)	[−36.08, 9.41]	18.00	0.33	0.21
TMT B accuracy	10	0.93 (0.90, 0.97)	0.90 (0.83, 0.97)	0.00 (0.00, 0.00)	[-0.34, 0.34]	6.00	0.18	0.30
TMT B:A ratio	10	1.92 (1.23, 2.67)	1.92 (1.44, 2.45)	0.03 (−0.90, 0.43)	[−0.66, 0.42]	24.00	0.72	0.08
**TAP Alertness**								
Mean RT w/o signal [ms]	10	299.0 (265.0, 387.5)	296.0 (260.8, 357.0)	15.0 (−42.0, 24.8)	[−30.68, 26.68]	25.00	0.80	0.06
Mean RT with signal [ms]	10	283.0 (267.8, 399.3)	269.0 (255.3, 346.5)	−10.0 (−26.3, 3.8)	[−46.84, 28.44]	14.00	0.31	0.23
**TAP Go-NoGo**								
Mean RT [ms]	10	498.5 (433.3, 542.5)	462.0 (398.3, 595.0)	−13.5 (−80.0, 56.3)	[-62.65, 54.45]	26.00	0.88	0.03
Errors	10	1.50 (1.00, 2.75)	2.00 (0.75, 2.25)	0.00 (−2.00, 1.00)	[−1.67, 0.87]	7.00	0.46	0.17
Omissions	10	0.00 (0.00, 1.00)	0.00 (0.00, 1.00)	0.00 (−0.25, 0.00)	[−0.76, 0.36]	1.50	0.41	0.18
**TAP incompatibility**								
Mean RT compatible [ms]	6	570.0 (509.0, 681.5)	603.0 (515.8, 785.0)	33.0 (6.8, 92.5)	[−25.83, 121.50]	18.00	0.12	0.46
Mean RT incompatible [ms]	6	721.5 (575.5, 807.3)	750.0 (575.3, 896.0)	35.0 (−10.0, 88.8)	[−15.06, 92.73]	16.00	0.25	0.33
Mistakes compatible	6	0.00 (0.00, 1.25)	0.00 (0.00, 1.00)	0.00 (−1.00, 0.25)	[−0.96, 0.62]	2.00	0.56	0.17
Mistakes incompatible	6	2.00 (0.00, 4.25)	1.50 (0.75, 3.00)	0.00 (−3.00, 1.75)	[−2.94, 2.60]	5.00	> 0.99	0.00
**MRT**								
Accuracy	9	0.58 (0.36, 0.61)	0.55 (0.46, 0.71)	0.13 (0.02, 0.19)	[−0.004, 0.18]	34.50	0.15	0.33
Mean RT of correct [ms]	9	1897.4 (1685.0, 2141.7)	1744.7 (1643.0, 1912.2)	−148.4 (−404.2, 154.8)	[−310.2, 46.6]	12.00	0.21	0.29

*N, number of participants included in test; T0, Baseline; T1, Post-Measurements; CI, confidence interval; T, test statistic of Wilcoxon signed rank test; RT, reaction time; w/o, without*.

*Values displayed in median (interquartile range); [s] seconds, [ms] milliseconds*.

According to the BREQ-3, the intrinsic motivation for general physical activity of the participants decreased over the course of the intervention with a trend toward significance and a medium effect size, while the other types of motivation for physical activity were maintained (Table 6). The SSK showed a significant decrease in identified motivation for physical activity with a medium to large effect size and a non-significant decrease in external motivation with a medium effect size. The SSK index did not change (Table 6).

**Table 6:** Results secondary motivational outcomes.

**Motivation outcomes**	**N**	**T0**	**T1**	**DifferenceT1–T0**	**95% CI**	**T**	**Asymptotic Significance**	**Effect size**
**BREQ-3**								
Intrinsic motivation	10	12.50 (10.00, 15.25)	12.50 (9.25, 13.00)	−1.00 (−3.00, 0.00)	[−2.17, 0.17]	4.00	0.09	0.38
Integrated motivation	10	12.50 (10.50, 16.00)	12.50 (8.50, 15.25)	0.00 (−3.25, 1.00)	[−2.57, 0.77]	8.00	0.31	0.23
Identified motivation	10	12.50 (12.00, 15.25)	14.00 (13.00, 14.25)	0.00 (−2.00, 2.00)	[−1.91, 1.51]	28.00	0.96	0.01
Introjected motivation	10	9.50 (1.75, 11.25)	7.00 (3.50, 10.25)	−0.50 (−4.25, 2.50)	[−3.08, 2.28]	19.50	0.72	0.08
External motivation	10	0.50 (0.00, 3.25)	2.00 (0.75, 3.00)	0.00 (−1.50, 3.00)	[−1.79, 2.79]	20.00	0.78	0.06
Amotivation	10	0.00 (0.00, 0.00)	0.00 (0.00, 0.25)	0.00 (0.00, 0.25)	[−0.53, 1.73]	3.00	0.18	0.30
**SSK-index**	10	14.5 (10.25, 21.50)	19.0 (10.00, 21.50)	1.0 (−2.50, 6.50)	[−1.95, 5.75]	38.00	0.28	0.24
Intrinsic motivation	10	14.50 (12.50, 16.50)	14.50 (12.00, 16.25)	0.00 (−1.25, 1.25)	[−1.51, 1.31]	10.00	0.92	0.02
Identified motivation	10	18.00 (17.00, 18.00)	17.00 (15.50, 18.00)	−1.00 (−2.25, 0.00)	[−2.19, −0.01]	2.50	**0.05**	0.44
Introjected motivation	10	9.50 (7.75, 14.75)	9.50 (8.00, 10.50)	0.50 (−5.50, 2.25)	[−5.43, 2.23]	15.00	0.67	0.09
External motivation	10	5.50 (3.00, 7.75)	4.00 (3.00, 5.00)	−1.00 (−3.75, 1.00)	[−3.99, 0.59]	10.50	0.15	0.32

*N, number of participants included in test; T0, Baseline; T1, Post-Measurements; CI, confidence interval; T, test statistic of Wilcoxon signed rank test*.

*Values displayed in median (interquartile range); in bold, significant at p < 0.05*.

## Discussion

This study investigated the feasibility of a rehabilitation approach using exergames and following an adapted skill-progression taxonomy in chronic stroke patients. Furthermore, possible effects of the rehabilitation approach on mobility, gait, cognitive functions, and motivation for physical activity in chronic stroke patients were explored. We found that the rehabilitation approach was feasible in terms of inclusion, adherence, compliance, motivation and satisfaction, and that, however, the attrition rate exceeded the pre-defined threshold. Each participant moved on an individual path through the taxonomy, however, the VAS ratings of perceived motor and cognitive task difficulty moved below the targeted range. This and the high adherence, compliance, motivation and satisfaction rates indicate that the adapted taxonomy enabled personalized progression, which, however, still has potential for improvement in achieving optimal task difficulty. A significant improvement was found for the TUG test, while none of the other motor and cognitive outcomes showed changes. However, medium to large effect sizes were found for improvements in the swing time on the affected side, the time needed for the TMT A and the accuracy in the TMT B as well as in the MRT. No changes in the degree of self-determination was observed for general physical activity in the BREQ-3 and future intention toward physical activity in the SSK scale.

### Primary Outcome—Feasibility

The inclusion, adherence and compliance rates as well as mean motivation and mean satisfaction during the trainings all reached or exceeded the thresholds set for feasibility (see Table 3, Figure 5), while the attrition rate was higher than the targeted threshold (see Table 3). This is in line with the results from other studies investigating exergame interventions in stroke patients and healthy older adults, where a similar recruitment process was implemented (Rozental-Iluz et al., 2016) and comparable adherence and compliance rates were observed (Anderson-Hanley et al., 2012; Schoene et al., 2013; Rebsamen et al., 2019; Burdea et al., 2020). Moreover, our results are in line with results in stroke and other neurological patients, where exergames and VR methods were found to be feasible for rehabilitation purposes and increased the motivation and satisfaction with rehabilitation interventions (Lange et al., 2010; Hamari, 2014; Cheok et al., 2015; Matallaoui et al., 2017; Dietlein et al., 2018; Mura et al., 2018; Garcia-Agundez et al., 2019; Wiley et al., 2020). The high attrition rate, however, indicates a problem with the exergame intervention itself. Other studies with exergame interventions reported lower attrition rates compared to this study (Fritz et al., 2013; Schattin et al., 2016). A possible reason therefore may be that this study took place during the time of the COVID-19 pandemic. This may have influenced the feasibility outcomes—in particular the inclusion and attrition rate—of this study as people were advised to shield and participants as well as investigators may have experienced limited allowance and emotional safety for contacts (Sloan et al., 2021). This is supported by the finding that one dropout was caused by the fear of getting infected with the Corona virus at the study center (see section Participants Overview). Moreover, several screened patients refused to participate, as they feared of an infection on the way or at the study center. However, the pandemic situation does not explain all dropouts, as one of them was caused by a training-related adverse event (see section Participants Overview). Exergames are generally reported to be safe (Cheok et al., 2015; Givon et al., 2016; Mura et al., 2018; Norouzi-Gheidari et al., 2019), which implies that this adverse event may be a rare case. Particularly, the participant concerned suffered an uncomfortable intensification of a pre-existing head tremor, possibly caused by the challenging simultaneous motor and cognitive actions required for the training. Therefore, the intervention could not be continued, however, it did not cause the head tremor but rather intensified the pre-existing condition. Nevertheless, such an adverse event should be prevented in a future study and how this may be done, is discussed below (section Future Directions).

### Secondary Outcomes—Strengths, and Limitations of the Adapted Taxonomy

A strength of the rehabilitation approach was the careful adaption of the taxonomy based on theoretical reasoning, that consistently applied principles of motor learning (Gentile, 2000). The personalization of the progression was a further strength, however, as can be concluded from the mean ratings of perceived task difficulty predominantly moving below the targeted range, the adapted taxonomy did not achieve optimal task difficulty throughout the intervention (Figure 6). Three limitations of the adapted taxonomy were identified. (1) The design of the taxonomy may have linked motor and cognitive progression in an improper way, as separate subjective ratings for motor and cognitive task difficulty and the separate progression on two axes rather decoupled motor and cognitive learning. This led to problems, particularly in patients who were cognitively rather fit and had motor impairments or, vice versa, struggled a lot with the cognitive tasks while being physically rather fit. We observed that in these cases, being unable to progress on one axis in the taxonomy, hindered progression on the other axis, which led to under-challenge of the stronger function. However, motor learning already contains a cognitive component, as it usually passes through three different stages, a cognitive phase, an associative phase, and an autonomous phase, in which the perception about motor and cognitive task difficulty will depend on the stage of learning an individual is in (Weaver, 2015). This would imply that it would be better to not decouple motor and cognitive components of motor-cognitive training and therefore, use one instrument for the determination of motor-cognitive task difficulty progression. However, currently there is no suitable instrument that can be recommended (Shishov et al., 2017). (2) Therefore, out of lack of a suitable alternative, subjective ratings of perceived motor and cognitive task difficulty were used to guide the progression in this study (Sweller, 2011). Subjective ratings bear the risk for over- or underestimation of the personal ability and performance. To prevent this, we used visual analog scales, as using a VAS has the advantage that the raters are not dependent on given options but can choose from a continuum to give their answer (Voutilainen et al., 2016). VAS may therefore result in greater responsiveness and be less vulnerable to bias from confounding factors (Pfennings et al., 1995; Voutilainen et al., 2016). Nevertheless, based on the obtained results of perceived task difficulty, we conclude that subjective ratings may not be the proper instrument to guide individualized progression in chronic stroke patients. Moreover, these subjective ratings may have been collected at the wrong time-point. Participants rated their perceived task difficulty at the very end of the session, after having completed the cool-down game. This time point was chosen to not interrupt the training session and therefore disturb the concentration and flow of the participants. Nevertheless, as this cool-down game was less difficult than the rest of the session (see section Intervention/Personalized Exergame Training), this may have influenced the ratings. The last game may have been most present in the thoughts of the participants when they rated their perceived task difficulty, which may have resulted in lower ratings than would have matched the actual perceived task difficulty during the main part. How therefore task difficulty should be progressed in a future trail is discussed below (section Future Directions). (3) Due to limited possibilities to adjust all games to all levels and a lack of games, which train memory functions, several skill sub-categories of the adapted taxonomy remained empty. This may have influenced the personalized progression, as in case an empty sub-category should have provided a game, this was substituted by a game from one of the surrounding sub-categories. As this was a feasibility study, this limitation was accepted, however, for a future study, it should be ensured that all sub-categories of the adapted taxonomy contain at least one game.

### Secondary Outcomes-Motor, Cognitive, and Motivational Outcomes

The secondary aim of this study was to get insight into possible treatment effects of the rehabilitation approach in chronic stroke patients. Therefore, we explored changes from before to after the intervention and compared the meaningfulness of change values and confidence intervals to comparable literature (Tables 4–6).

The significant improvement in the TUG from pre- to post-measurements with a medium to large effect size is in line with other studies using exergames in chronic stroke patients, which found significant improvements in the TUG and comparable changes and confidence intervals to our results (Singh et al., 2013; Cheok et al., 2015). These changes in the TUG were significantly greater in groups with additional exergame training compared to the control groups receiving standard care, which corresponds with the conditions of this study (Cheok et al., 2015). We found a medium effect size for the non-significant decrease in swing time on the affected side, while no change and a very small effect size was observed for swing time on the unaffected side. A review on spatio-temporal gait parameters in chronic stroke patients found that the changes in swing time are more often observed on the paretic side compared to non-paretic side (Wonsetler and Bowden, 2017). Significant changes were generally greater compared to our findings. Swing time is an important gait parameter in stroke patients as it is associated with single limb support and postural stability (Chisholm et al., 2014). Furthermore, we found a small to medium effect size for the improvement in the asymmetry index, which is a parameter of special interest in chronic stroke patients (Patterson et al., 2008). A temporal asymmetry index between 0.9 and 1.1 is considered normative, while 1.1–1.5 indicates mild asymmetry and >1.5 severe asymmetry (Patterson et al., 2008). The median asymmetry index of the participants moved within the normative range closer to 1. Little is yet known about possible effects of exergame training on gait symmetry in stroke patients, however, high intensity exercise has been found to affect gait symmetry in stroke patients. A meta-analysis reported a trend toward significance (Luo et al., 2019) and our study seems to support this finding. The results of these motor outcomes seem a promising finding indicating that these chronic individuals still have room to improve their mobility and gait if adequate intervention content is provided. The TUG, swing time and the asymmetry index may serve as possible outcomes for a future study.

Among the cognitive outcomes, we found medium to large effect sizes for reduced time needed for the TMT A and accuracy in the TMT B and the MRT. These results are in line with two reviews, which found beneficial effects of exergames on cognitive functions including global cognition, executive functions and visuo-spatial perception in neurological patients and any population, respectively (Stanmore et al., 2017; Mura et al., 2018). In chronic stroke patients, highly significant improvements in time for TMT A and B were found after a dual-task training intervention (Park and Lee, 2019). This gives rise to the presumption that attentional processing speed and cognitive flexibility may be affected by motor-cognitive interventions in general and therefore may be recommendable outcomes for a future randomized controlled trial (RCT). Furthermore, we found moderate effect sizes for increased reaction times in the TAP Incompatibility. The TAP Incompatibility and the MRT, however, are intended to be completed with two hands, which more than half of the stroke patients included in this study were not able to do due to paretic arms. Therefore, some skipped the tests and a few used one hand to operate both buttons. This may have influenced the results, even though the procedures from the baseline measurements were repeated exactly in the post measurements to ensure comparability of the results between the two time points. Due to operational limitations for these tests, we do not recommend them for being used in a future RCT despite the promising effect sizes. To cover attentional performance on incompatible stimuli and mental rotation, other tests should be chosen, which do not require action of both hands.

In the BREQ-3, we found a trend toward significance and a medium effect size for a decrease in intrinsic motivation for general physical activity, while the other motivation sub-types and a motivation were maintained. In the SSK scale, identified motivation for future physical activity decreased significantly and showed a medium to large effect size, while the SSK index showed no change. These results are in line with a meta-analysis on the motivational effects of exergames, which found no difference between exergames and alternative instructional methods (Wouters et al., 2013). This stands in contrast to literature, which states that exergames are expected to increase self-determination of exercise (Lange et al., 2010; Matallaoui et al., 2017; Sailer et al., 2017) and have been found to be superior in improving intrinsic motivation compared to a non-gaming condition (Fitzgerald et al., 2010). It is argued that specific gaming elements such as points, badges and leader boards are important for raising self-determination for exercise (Sailer et al., 2017). Such gaming elements not being present or not being noticed enough by the trainees may be an explanation for lower self-determined motivation during exergame interventions (Sailer et al., 2017). This aspect may apply for the games used in this study, as several participants expressed the wish for more feedback during and after the games. These considerations suggest to further investigate self-determined motivation for exercise in future trials with exergames and to improve the choice and implementation of the motivational elements in exergames (Matallaoui et al., 2017).

### Implications, Strengths, and Limitations of This Study

To our knowledge, this is one of the first studies to assess the feasibility of a rehabilitation approach using exergames and following a skill-progression taxonomy in chronic stroke patients. Therefore, this study contributes to the advancement of long-term care for stroke survivors, addressing motor and cognitive impairments. Progressing individually through the taxonomy based on current skills may be a motivating factor for the trainees that taps on the things they are currently able to do. In this scenario, the progression through the taxonomy may be based on patterns of recovery shared by similar patients. Future studies applying larger samples may be well-advised to also consider data mining approaches with the aim to uncover patterns of recovery (Marcano-Cedeño et al., 2013; Chu et al., 2020). Strengths of this study were the clear and pre-defined feasibility protocol, the rather long study duration for a feasibility study and the combination of motor and cognitive outcomes. Nevertheless, several limitations should be considered when reading this study. (1) As being a feasibility trial, this study was designed without a control group, with a total N and treatment duration sufficient to evaluate feasibility but not treatment effects. Therefore, the results in the secondary outcomes must be interpreted with caution and not generalized to other populations. Nevertheless, the derived data and effect sizes can be used for future sample size calculations. (2) The study sample was rather high functioning in mobility and cognitive assessments, highly educated and all but one participant attended regular physiotherapy (see baseline values, Table 2), which may have been a reason for the few changes observed in the secondary outcomes. As this was a feasibility study, the training principle “initial value” (Hoffman, 2002), which relates to the baseline performance of a trainee, was not considered as an inclusion criterion. Particularly, this did not hinder the primary outcome of this study—feasibility—to be evaluated. Nevertheless, for a future study, further eligibility criteria regarding “initial values” may be considered to increase the potential for detecting treatment effects (see section Future Directions). (3) Moreover, as participants in this study were recruited in a physiotherapy center and a senior home, the study team did not have contact to the participants' clinicians. Therefore, several diagnostic information, which is traditionally included in reports of studies conducted in a stroke population (e.g., ischemic/haemorrhagic stroke, affected territory) is missing in this report. This was considered acceptable as this study primarily investigated feasibility. In a future RCT, however, with the aim to investigate treatment effects, this information should be included (Kwakkel et al., 2017). (4) One participant was not able to perform the computer-based version of the TMT, as they were not proficient enough with a computer mouse (so that the test performance would probably have been limited by their speed to use the mouse and not by cognitive skills to accomplish the task). To prevent missing data, we administered a paper-and-pencil version of the test. This should, however, be prevented in a future trial, therefore careful considerations regarding the choice of the test version in the targeted study population and sample size should be undertaken. (5) Several assessments exhibited missing data, which were caused by reasons described above (section Secondary Outcomes). Several participants were not able to perform the cognitive tests, which required use of both hands. This should be prevented in a future study by choosing cognitive assessments, which can be completed with one hand. (6) A possible effect of the two study centers could not be investigated, as only one participant was finally recruited in the senior home and was therefore the only participant, who did not receive other therapy or attend therapeutical activities besides the study sessions.

### Future Directions

As the rehabilitation approach was feasible with minor modifications and promising results were found for some outcomes, which are relevant for stroke rehabilitation, we recommend the conduction of a well-designed randomized-controlled trial to investigate the effects of the rehabilitation approach using exergames and following a skill-progression taxonomy in comparison to a control intervention. To achieve appropriate credible training effects in such a trial, an appropriate sample size should be achieved, and the intervention duration should be increased to at least 12 weeks (Lauenroth et al., 2016). To account for the training principle “initial value,” further eligibility criteria regarding the motor and cognitive impairments of the participants may be considered (e.g., particularly participants with mild to moderate cognitive impairment may be included). To ensure the safety of the intervention, patients experiencing any problems during the use of VR or during challenging simultaneous motor and cognitive action should be excluded. To support this, a familiarization session with the training system for all interested participants may be helpful to foresee adverse reactions to the training. Moreover, before conducting a following study, the taxonomy should be revised. Specifically, we recommend expanding and refining the taxonomy to include a greater range of different motor and cognitive levels by adding more games, in particular for memory functions. Moreover, it may be recommendable to couple motor and cognitive progression more closely, e.g., by using a motor-cognitive parameter for guiding the progression instead of two separate outcomes for motor and cognitive task difficulty, respectively. To improve the progression rules, objective measures may be identified to monitor task difficulty during the training and replace the subjective ratings. Possible parameters therefore may be the reaction times to the stimuli in the games or the accuracy achieved in the cognitive tasks. Moreover, in future research, it may be preferable to combine external load measures with measures approximating internal load (Paas et al., 2003; Herold et al., 2020a). Conceivalbe solutions may be measuring eye activity such as cognitive pupillary response (Paas et al., 2003), galvanic skin response (Jaiswal et al., 2020) or functional near-infrared spectroscopy (fNIRS) (Herold et al., 2020b). Furthermore, the possibility to transfer the intervention into a tele-rehabilitation setting may be promising as stroke patients but also other neurological patients may generally be limited in their ability to reach study centers. Additionally, this would also improve the access to rehabilitation interventions during situations such as the COVID-19 pandemic or COVID-19 patients who are quarantined (Paneroni et al., 2021).

## Data Availability Statement

The original contributions presented in the study are included in the article/Supplementary Material, further inquiries can be directed to the corresponding authors.

## Ethics Statement

The studies involving human participants were reviewed and approved by the Ethical Committee of the ETH Zürich (approval no. 2019-N-180). The patients/participants provided their written informed consent to participate in this study.

## Author Contributions

SH developed the taxonomy, the methodology and study protocol under the supervision of EB and RK. JH facilitated the recruitment process by providing contacts to the physiotherapy center in Zürich. SH performed data acquisition and analysis, the first interpretation of the data, the writing of the first version of the manuscript and manuscript revision. JH, EB, and RK were involved in in-depth interpretation of the data, revision of the scientific content and manuscript revision. The final manuscript was read and approved by all authors.

## Funding

This work was enabled by resources of the University Hospital of Zurich and ETH Zürich. The study device in one study center was provided by Dividat AG, however, Dividat AG was not involved in any decisions regarding study-related decisions including the determination of the study design, the collection, management, analysis, and interpretation of the data, and writing this study report.

## Conflict of Interest

The authors declare that the research was conducted in the absence of any commercial or financial relationships that could be construed as a potential conflict of interest.

## Publisher's Note

All claims expressed in this article are solely those of the authors and do not necessarily represent those of their affiliated organizations, or those of the publisher, the editors and the reviewers. Any product that may be evaluated in this article, or claim that may be made by its manufacturer, is not guaranteed or endorsed by the publisher.

## Footnotes

^1^Timed-up-and-go test. Available online at: https://www.physio-pedia.com/Timed_Up_and_Go_Test_(TUG).
