# Supplementary material for: Personalized Motor-Cognitive Exergame Training in Chronic Stroke Patients—A Feasibility Study
Source: Front Aging Neurosci. 2021 Oct 20;13:730801. doi: 10.3389/fnagi.2021.730801 (PMC8565485; doi:10.3389/fnagi.2021.730801)
Supplement: Supplementary file 1 [file Table_1.docx]

Date: Participant-ID:

After-training Ratings

Please mark the level of your motivation and satisfaction with today’s training on the lines below.


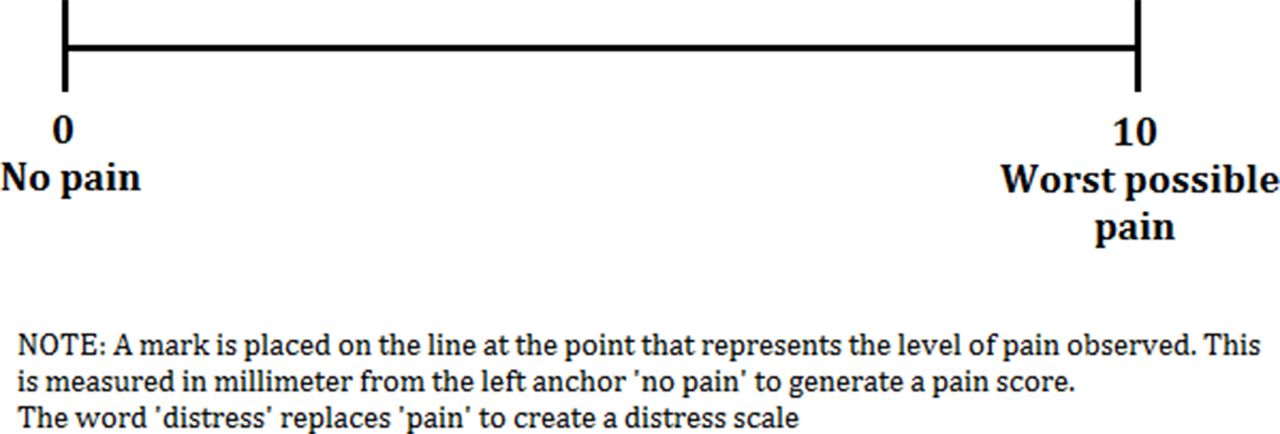
How motivated were you for today’s training?

Not at all

Totally

How satisfied were you with today’s training?


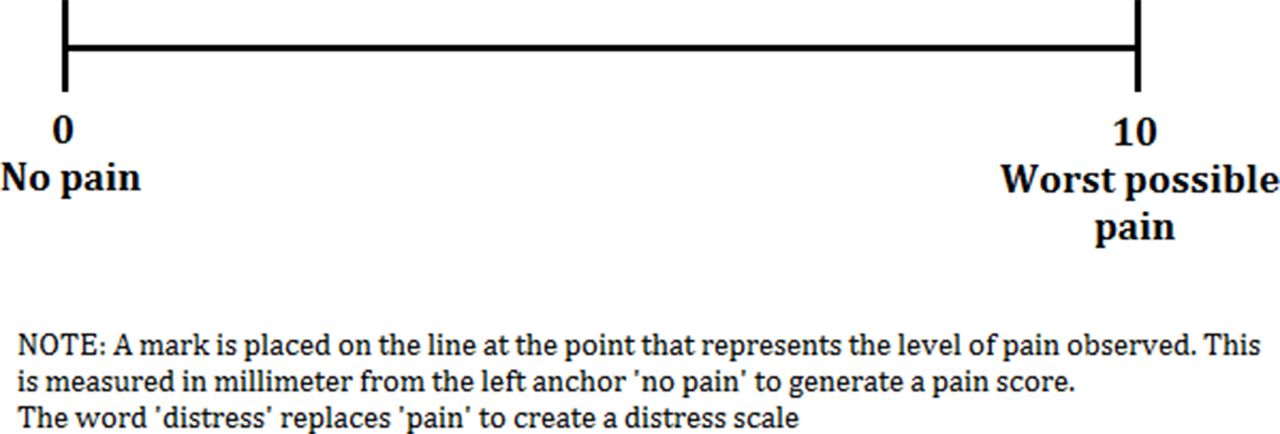


Totally

Not at all
