# Supplementary material for: Personalized Motor-Cognitive Exergame Training in Chronic Stroke Patients—A Feasibility Study
Source: Front Aging Neurosci. 2021 Oct 20;13:730801. doi: 10.3389/fnagi.2021.730801 (PMC8565485; doi:10.3389/fnagi.2021.730801)
Supplement: Supplementary file 2 [file Table_2.docx]

# Supplementary Table 1: Feasibility Criteria and Protocol

| **Feasibility Criteria** | **Definitions** | | **Thresholds for Feasibility** |
| --- | --- | --- | --- |
| Recruitment rate*  Adherence rate*  Compliance rate*  Attrition rate*  Motivation rate**  Satisfaction rate** | = (pre-)screened people / enrolled participants  = attended / offered training sessions  = mean attended / mean offered training time per session  = drop-outs / enrolled participants  = mean motivation / maximal motivation on VAS  = mean motivation / maximal motivation on VAS | | ≥ 15 %  ≥ 80 %  ≥ 80 %  ≤ 15%  ≥ 60%  ≥ 60% |
| **Possible Scenarios:** | | **Consequences** | |
| a) All feasibility criteria met. | | *Intervention is feasible; RCT can be conducted without modifications.* | |
| b) Recruitment rate too low. | | *Evaluate the reasons for failed recruitments:*  *- in case of intervention-unrelated reasons only, reconsider recruitment procedure, but conduct the intervention without modifications in the RCT*  *- in case of intervention-related reasons, implement appropriate modifications to the intervention for the RCT* | |
| c) Attrition rate too high. | | *Evaluate reasons for dropouts:*  *- in case of training-unrelated dropouts only, continue with RCT without modifications*  *- in case of training-related dropouts, modifications must be undertaken to hinder similar events in the RCT*  *- in case of a drop-out due to a training-related serious adverse event, the RCT must not be conducted* | |
| d) Adherence, compliance, motivation, satisfaction rates too low. | | *Evaluate reasons for unsatisfactory results and implement according to minor modifications for the RCT.* | |
| e) ≥ 3/6 feasibility criteria not met. | | *Evaluate reasons and implement major modifications for the RCT.* | |
| f) ≥ 5/6 feasibility criteria not met. | | *RCT must not be conducted.* | |

VAS, Visual analogue scale
* based on guidelines from Nyman et al.
** established from comparable studies, Van Beek et al. 2019, Bernadoni et al. 2019, Spildooren et al. 2019
